# Mitochondrial apurinic/apyrimidinic endonuclease 1 enhances mtDNA repair contributing to cell proliferation and mitochondrial integrity in early stages of hepatocellular carcinoma

**DOI:** 10.1186/s12885-020-07258-6

**Published:** 2020-10-07

**Authors:** Veronica Bazzani, Arianna Barchiesi, Dorota Radecka, Riccardo Pravisani, Antonio Guadagno, Carla Di Loreto, Umberto Baccarani, Carlo Vascotto

**Affiliations:** 1 Department of Medical Area, University of Udine, P.le Massimiliano Kolbe 4, 33100 Udine, Italy; 2 Department of Medicine, General Surgery and Transplantation, Academic Hospital (ASUIUD), University of Udine, Udine, Italy; 3 Department of Medicine, Institute of Pathology, University of Udine, Udine, Italy; 4 Pathology Unit, IRCCS Ospedale Policlinico “San Martino”, Genoa, Italy

**Keywords:** Hepatocellular carcinoma, APE1, MIA pathway, Mitochondria, mtDNA damage, Base excision repair pathway

## Abstract

**Background:**

Hepatocellular carcinoma (HCC) is the leading cause of primary liver cancers. Surveillance of individuals at specific risk of developing HCC, early diagnostic markers, and new therapeutic approaches are essential to obtain a reduction in disease-related mortality. Apurinic/apyrimidinic endonuclease 1 (APE1) expression levels and its cytoplasmic localization have been reported to correlate with a lower degree of differentiation and shorter survival rate. The aim of this study is to fully investigate, for the first time, the role of the mitochondrial form of APE1 in HCC.

**Methods:**

As a study model, we analyzed samples from a cohort of patients diagnosed with HCC who underwent surgical resection. Mitochondrial APE1 content, expression levels of the mitochondrial import protein Mia40, and mtDNA damage of tumor tissue and distal non-tumor liver of each patient were analyzed. In parallel, we generated a stable HeLa clone for inducible silencing of endogenous APE1 and re-expression of the recombinant shRNA resistant mitochondrially targeted APE1 form (MTS-APE1). We evaluated mtDNA damage, cell growth, and mitochondrial respiration.

**Results:**

APE1’s cytoplasmic positivity in Grades 1 and 2 HCC patients showed a significantly higher expression of mitochondrial APE1, which accounted for lower levels of mtDNA damage observed in the tumor tissue with respect to the distal area. In the contrast, the cytoplasmic positivity in Grade 3 was not associated with APE1’s mitochondrial accumulation even when accounting for the higher number of mtDNA lesions measured. Loss of APE1 expression negatively affected mitochondrial respiration, cell viability, and proliferation as well as levels of mtDNA damage. Remarkably, the phenotype was efficiently rescued in MTS-APE1 clone, where APE1 is present only within the mitochondrial matrix.

**Conclusions:**

Our study confirms the prominent role of the mitochondrial form of APE1 in the early stages of HCC development and the relevance of the non-nuclear fraction of APE1 in the disease progression. We have also confirmed overexpression of Mia40 and the role of the MIA pathway in the APE1 import process. Based on our data, inhibition of the APE1 transport by blocking the MIA pathway could represent a new therapeutic approach for reducing mitochondrial metabolism by preventing the efficient repair of mtDNA.

## Background

Hepatocellular carcinoma (HCC) represents between 75 and 85% of primary liver cancers [1]. It is the sixth most prevalent malignancy and the fourth leading cause of cancer-related deaths around the world, with about 841,000 new cases and 782,000 deaths annually [1].

In patients with end-stage liver disease and early stage HCC (defined by the Milan criteria according to the EASL guidelines), liver transplantation is considered to be the treatment option with the highest curative effect [2]. However, limitations of liver transplantation apart from donor shortage include the risk of dropout while waiting (4% per month) and the perioperative mortality rate [3]. Liver resection represents an alternative cure, although it is only feasible in fewer than 20% of patients because of local spread and severity of pre-existing cirrhosis [4]. If HCC is unresectable due to multinodular presence or extrahepatic spread, local treatments such as radiofrequency ablation, percutaneous ethanol injection, and systemic therapies are the only options [4]. Surveillance of individuals at specific risk of developing HCC, early diagnostic markers, and new therapeutic targets are essential to obtain a reduction in disease-related mortality.

Mitochondria are essential organelles with numerous functions in cellular metabolism and homeostasis, among which is the energy conversion to ATP [5]. Although the majority of the mitochondrial proteome is composed of nuclear encoded proteins, each mitochondrion possesses multiple copies of mitochondrial DNA (mtDNA) containing 37 genes, all of which are essential for normal mitochondrial function [5]. Because of its proximity to the electron transport chain (ETC), mtDNA is subject to damage more than nuclear DNA (nDNA). Indeed, the major source of endogenous reactive oxygen species (ROS) is the electrons that have escaped from the ETC [6–8]. The most extensively studied and most common lesion caused by exposure to ROS is 8-oxoguanine (8-oxoG). 8-oxoG has been found to be a mutagenic lesion: mispairing of 8-oxoG with adenine results in a G-C to A-T transversion mutation during the subsequent round of replication. This particular mutation is present in significantly higher levels in mtDNA [9, 10]; moreover, the damage has been described as more extensive, persistent, and longer lasting than nuclear DNA damage in human cells following oxidative stress [11]. To cope with damage formatting and to maintain their functionality, mitochondria are equipped with efficient mechanisms aimed at repairing occurring lesions. One of the main pathways present in this organelle is the mitochondrial BER pathway (mtBER), which is involved in repairing non-helix-distorting base lesions and contributing to maintenance of DNA stability [12]. The presence of several components of this pathway has been documented in mitochondria, such as OGG1, UDG, MUTY and NTH [13–17] as well as DNA ligase III [18, 19] and APE1 [20, 21].

APE1’s localization is eminently nuclear, although this protein is also present within the mitochondrial matrix. APE1 is a key element of the BER pathway, both in the nucleus and mitochondria, and aside from its endonuclease activity, the protein is also a redox factor associated with the transcriptional regulation of gene expression [22]. Information about APE1’s subcellular trafficking is still scanty and incomplete. What is known is that the protein presents a bipartite nuclear localization signal (NLS) within its N-terminal domain that directs the protein within the nucleus [23]. Concerning the mitochondrial translocation, it has been demonstrated that APE1 uses the TOM pore complex to pass the outer membrane [24] and reach the mitochondrial inner membrane space (IMS), where it becomes the substrate of the MIA pathway [25]. However, the mechanics explaining how the protein is further moved into the matrix, and the molecular events conveying the protein into the nucleus rather than mitochondria, are still unknown.

Due to its involvement in essential cellular processes such as genome stability and gene expression regulation, the importance of APE1 in human pathologies such as cancers, neurological diseases, and age-associated disorders is not surprising. Many studies have shown that APE1 is overexpressed in a variety of cancers, suggesting a possible prognostic significance and therapeutic target for this protein [26–29]. Data from different laboratories support a correlation between the increased expression levels of APE1 and HCC progression [30–33] and uphold the hypothesis that APE1 loss of expression suppresses proliferation and migration [34–36]. This delineates APE1 as both a molecular marker and a druggable target in HCC. However, apart from its overexpression, an uncanonical cytoplasmic positivity of APE1 in HCC has been reported to correlate with a lower degree of differentiation and shorter survival rate [37, 38]. The aim of this study is to fully investigate, for the first time, the role of the mitochondrial form of APE1 in HCC.

## Methods

### Samples of human tumor tissue specimens and adjacent non-tumor tissues

Samples of paired HCC and adjacent non-tumor liver tissues from patients undergoing HCC resection were obtained from the Department of Medicine, General Surgery and Transplantation of the University of Udine, Udine, Italy (Table 1). None of the patients had received any local or systemic anticancer treatments before the surgery. Diagnosis of HCC was performed in all cases by preoperative imaging (CT or MRI scan) or by liver biopsy when requested. Hepatic serology, α-fetoprotein (AFP), routine laboratory assessment of liver and renal function were also performed. The presence of suspected (based on radiologic features) neoplastic main branch portal thrombosis was considered a contraindication to surgery [39].

After hospital discharge all patients were followed up and monitored for tumor recurrence by monthly assessments of serum AFP and by US, CT or MRI scan every 3–6 months. HCC recurrence was in all cases diagnosed by CT or MRI imaging according to international guidelines [2]. This study was approved by the Unique Regional Ethic Committee, August 24^,^ 2019, Protocol number 18659, and informed consent was obtained from each patient.

**Table 1:** Patients’ Demographic and Clinical Characteristics, Tumor Features, and Outcome

Gender (M:F)	15:5
Age (years)	73 [62–77]
HBV positivity (%)	1 (5%)
HCV positivity (%)	6 (30%)
NASH (%)	3 (15%)
Alcohol abuse (%)	7 (35%)
MELD score	7 [6–10]
AFP, (ng/mL)	7.5 [3.2–23.5]
CEA, (ng/mL)	1.7 [1.1–3.3]
CA 19.9 (UI/mL)	16.9 [9.9–19.3]
Tumor number (%)	
- 1	14 (70%)
- 2	4 (20%)
- 3	2 (10%)
Satellites lesions (%)	3 (15%)
Tumor max diameter (cm)	4.3 [3–10.8]
Edmonson-Steiner grading (%)	
- G1	4 (20%)
- G2	12 (60%)
- G3	4 (20%)
Microvascular invasion (%)	7 (35%)
Tumor staging (%)	
- T1	7 (35%)
- T2	7 (35%)
- T3	3 (15%)
- T4	3 (15%)
Tumor recurrence (%)	10 (50%)
Recurrence time, (months)	25.4 [7.4–33.5]

Continuous variables are expressed by median [interquartile range], Categorical variables by percentage

*AFP* alphafetoprotein; *CA 19.9* Carbohydrate Antigen 19.9, *CEA* carcinoembryonic antigen, *HBV* hepatitis B virus, *HCV* hepatitis C virus, *NASH* non alcoholic steatohepatitis, *MELD* Model for end-stage liver disease

### Immunohistochemical analysis

In the registry of the Pathology Department of University Hospital of Udine, Udine, Italy, a total of 20 patients who had a pathological diagnosis of hepatocellular carcinoma between January 2015 and December 2017, were identified. For each case, a single pathologist looked at slides stained with hematoxylin-eosin to evaluate the histological grading of HCC according to the Edmondson and Steiner criteria. Another slide (not stained) was used for immunohistochemical analysis. Each slide included both the tumor and not neoplastic liver (cirrhosis or normal liver).

Immunoistochemical detection of APE1 was performed by immunohistochemistry using the anti-APE1 mouse monoclonal antibody as the primary antibody (Novus Biologicals, Cambridge, England). The slides were deparaffinized and rehydrated (Xylene: three washes for 5 min each; 100% Ethanol: two washes 10 min each; 95% Ethanol: two washes 10 min each; 70% Ethanol: two washes 10 min each; 50% Ethanol: two washes 10 min each; distilled water: two washes for 5 min). Using microwave, the tissue sections were brought to boil in 10 mM sodium citrate buffer (pH 6.0) and then maintained at a sub-boiling temperature for 10 min. The tissue sections were quenched with 3.0% hydrogen peroxide in methanol for at least 15 min to block endogenous peroxidase activity. To permeabilize the cells, the tissue sections were washed with 1% animal serum in PBS with 0.4% Triton X-100 (PBS-T). Then, the tissue sections were incubated with 5% animal serum in PBS-T for 30 min at room temperature to block any non-specific binding. The primary antibody (diluted 1:200; Novus Biologicals, Cambridge, England) was added and the tissue sections were incubated for 12 h at room temperature and then at 4 °C overnight. A DAKO REAL EnVision Rabbit/Mouse (K5007) was used as a second antibody. Horseradish peroxidase activity was detected using DAKO REAL 3,3′-diaminobenzidine + chromogen (K5007) as substrate for 3 min in accordance with the manufacturer’s instructions. Sections were counter-stained with hematoxylin with a cover slip. We consider the reaction for APE1 positive when a dense, homogeneous brown staining is identified in the nucleus of hepatocyte and when a granular brown staining is identified in the cytoplasm of hepatocyte.

### Cell culture

Stable HeLa clones for inducible silencing of endogenous APE1 and re-expression of recombinant shRNA resistant APE1 WT and mitochondrially targeted APE1 (MTS-APE1) were obtained as previously described [40, 41]. MTS-APE1 resistant sequence was designed substituting the N-terminal sequence involved in the nuclear localization of the protein with the well-characterized MTS sequence of manganese-superoxide dismutase (MLSRAVCGTSRQLAPALGYLGSRQ) [23, 24]. Expression and localization of the recombinant protein were confirmed by WB (Fig. 2a). APE1 silencing was induced by addition of doxycycline to the cell culture medium at a final concentration of 1 μg/mL for 10 days.

### Nuclei and mitochondria isolation from human HCC tissue specimens

After collection, all procedures were carried out at 4 °C and in the presence of protease inhibitors to avoid proteins degradation. Fresh samples were finely minced, suspended in 5 mL of Isolation Buffer (IB) [10 mM Tris/MOPS, 1 mM EGTA/Tris, 200 mM Sucrose], and homogenized. Then, sample were centrifuged at 70 x *g* for 3 min to remove non-homogenized tissue. Supernatant was further centrifuged at 600 x *g* for 10 min to separate nuclear (pellet) and mitochondrial (supernatant) fractions. Nuclei were washed in T1 solution [10 mM HEPES pH 7.9, 0.1 mM EDTA pH 8.0, 10 mM KCl, 0.1 mM MgCl_2_] and then lysed in T2 solution [10 mM HEPES pH 7.9, 0.1 mM EDTA pH 8.0, 400 mM NaCl, 1.5 mM MgCl_2_, 5% glycerol] for 20 min on ice. After centrifugation at 14.000 x *g* for 20 min, the supernatant, accounting for nuclear protein extract, was collected. Mitochondria were centrifuged at 7.000 x *g* for 10 min, washed once in IB buffer, and then resuspended in IB buffer. Nuclear and mitochondria protein extracts were then quantified using Bio-Rad protein assay reagent (Bio-Rad).

### mtDNA damage measurement by quantitative PCR in patients’ samples

mtDNA isolation and damage measurement were performed as previously described by Barchiesi et al. [42]. Briefly, mtDNA was extracted by patients isolated mitochondria from non-tumor or HCC sample using a plasmid isolation kit [43] and quantified by Quant-iT™ PicoGreen™ dsDNA Reagent (Invitrogen). Q-PCR was performed on each sample to amplify a 16 ∼ Kbp fragment, using the following primers: FOR 5′-TCT AAG CCT CCT TAT TCG AGC CGA-3′ and REV 5′- CCA TCC AAC ATC TCC GCA TGA TGA AA-3′. Fluorescence readings of the Q-PCR reactions were quantified in triplicate with Quant-iT™ PicoGreen™ dsDNA Reagent (Invitrogen) and then averaged for each sample. Blank value was subtracted and the ratio of the fluorescence readings obtained for the tumor tissue to those of the distal section determined the relative amplification of the mtDNA for each patient sample. Relative mtDNA damage was then expressed as the inverse of this relative amplification.

### Preparation of total cell extracts and subcellular fractionation

To prepare total protein extracts, cells were harvested by trypsinization and centrifuged at 250 x *g* for 5 min at 4 °C. The pellet was washed once with cold PBS and then resuspended in Lysis buffer [50 mM Tris-HCl pH 7.5, 150 mM NaCl, 1 mM ethylenediaminetetracetic acid (EDTA), 1% (vol/vol) Triton X-100, protease inhibitor cocktail (Sigma), 0.5 mM phenylmethylsulfonyl fluoride (PMSF)] at a cell density of 10^7^ cells/mL, incubated on ice for 30 min, and centrifuged at 20.000 x *g* for 20 min at 4 °C. The supernatant was collected as total cell lysate (TCE). For subcellular fractionation, cells were scraped in PBS, collected, and centrifuged at 250 x *g* for 5 min. Then, the pellet was suspended at a cell density of 100 mg/mL in Mitochondrial Isolation Buffer (MIB) [20 mM HEPES pH 7.6, 1 mM EDTA, 220 mM Mannitol, 70 mM Sucrose] supplemented with 2 mg/mL Bovine Serum Albumin (BSA). Cells were mechanically broken using a 7 mL dounce homogenizer (Wheaton), centrifuged at 650 x *g* for 10 min at 4 °C. The pellet was conserved to prepare nuclear subfraction. Supernatant collected was centrifuged at 14.000 x *g* for 15 min at 4 °C. Isolated mitochondria were washed with MIB supplemented with 2 mg/mL BSA and 1 M KCl and centrifuged as before. A last wash was performed using MIB without BSA, and then mitochondria were resuspended in MIB and considered as mitochondrial protein extract (MCE). In parallel, nuclei were resuspended in T1 solution [10 mM HEPES pH 7.9, 10 mM KCl, 0.1 mM MgCl_2_, 0.1 mM EDTA70, 2 mM PMSF] and centrifuged at 1.000 x *g* for 15 min at 4 °C. This step was performed twice followed by nuclei resuspension in T2 lysis buffer [20 mM HEPES pH 7.9, 420 mM NaCl, 1.5 mM MgCl_2_, 0.1 mM EDTA70, 5% glycerol, 2 mM PMSF]. Samples were incubated on ice for 20 min and centrifuged at 20.000 x *g* for 20 min at 4 °C. Supernatant represented the nuclear protein fraction (NCE).

Protein concentration was determined using Bio-Rad protein assay reagent (Bio-Rad). Subfractions purity was evaluated by Western blot analysis using LSD1 and ATP5A as nuclear and mitochondria markers, respectively, to exclude the presence of cross contaminations between the two organelles.

### Western blot analysis

Total (TCE), nuclear (NCE) or mitochondrial (MCE) protein extracts were quantified via Bradford assay and then reported amount were separated onto 12% SDS-PAGE. Then, proteins were transferred into a nitrocellulose membrane (Sartourius Stedim Biotech S.A.). Saturation of the membranes was performed for 1 h at room temperature using 5% non-fat dry milk in TBS-T [1XTBS supplemented with 0.1% Tween 20], followed by primary antibody incubation overnight at 4 °C [anti-APE1: 1:1.000 monoclonal (Novus); anti-Mia40: 1:500 polyclonal (costumed produced by APS Antibody Production Services); anti-FLAG: 1:1.000 monoclonal (Sigma); anti-ATP5A: 1:2.000 monoclonal (Abcam); anti-LSD1: 10.000 polyclonal (Abcam); anti-Actin: 1:2.000 polyclonal (Sigma Aldrich)]. Membranes were washed three times for 5 min with TBS-T, incubated for 2 h with the secondary antibody, and washed again for three time. The signal was detected with the Odyssey CLx scanner (Li-Cor Bioscience) and densitometric analysis was performed with ImageStudio software (Li-Cor Bioscience). In accordance with our Digital Image Integrity Policy uncropped Western blot images have been included as Supplementary Materials.

### DNA extraction and mtDNA damage analysis in cell lines

DNA was extracted using Qiagen genomic-tip 20/G and following manufacturer’s indications. After isolation DNA was precipitated overnight with isopropanol, and then 10 μg were digested with Formamidopyrimidine DNA Glycosylase (Fpg) enzyme at 37 °C for 30 min to remove damaged bases leaving an abasic (AP) site. Fpg was inactivated at 60 °C for 10 min and DNA was precipitated overnight, resuspended in 50 μL of Tris-EDTA buffer pH 8.0. Quantification was determined with Quant-iT™ PicoGreen™ dsDNA Reagent (Invitrogen), according to manufacturer’s instructions and DNA concentration was adjusted to 3 ng/μL.

mtDNA lesions were quantified by Q-PCR, using the following primers: Mitolong Forward: 5′-TCT AAG CCT CCT TAT TCG AGC CGA-3′ and Mitolong Reverse: 5′-TTT CAT CAT GCG GAG ATG TTG GAT GG-3′ which amplified an 8.9 Kbp mitochondrial fragment; Mitoshort Forward: 5-CCC CAC AAA CCC CAT TAC TAA ACC CA-3′ and Mitoshort Reverse: 5′-TTT CAT CAT GCG GAG ATG TTG GAT GG-3′ which amplified a 221 bp mitochondrial fragment. DNA was amplified using Platinum™ SuperFi™ DNA Polymerase (Invitrogen) using the following protocol: 2 min at 94 °C, 18 cycles of denaturation for 15 s at 94 °C, annealing for 10 s at 66 °C, extension for 5.30 min at 68 °C for the 8.9 Kb fragment or annealing 45 s at 60 °C and extension for 45 s at 72 °C for the 221 bp fragment. A final extension for 10 min at 68 or 72 °C was performed for each fragment. To ensure quantitative conditions a sample with the 50% of template amount was included in each amplification and, as negative control, a sample without the template were used. PCR products were quantified in triplicate by using Quant-iT™ PicoGreen™ dsDNA Reagent (Invitrogen). The Mitoshort fragment was used to calculate the relative amount of mtDNA copies and to normalize the lesions frequency calculated with the Mitolong fragment [43].

### Clonogenic assay

For the clonogenic assay, 500 cells were plated the day before the beginning of the silencing. After 10 days, cells were stained with 0.5% (wt/vol) methyl violet. Four biological replicates were preformed and for each replicate four 10 cm^2^ plates per clone were analyzed. Plates were imaged using a live scanner (GE Healthcare). The analysis was performed using a modified CellProfiler pipeline for colonies counting [44]. Briefly, the pipeline used was based on four steps: background correction, identification of the single plate, colony detection, and measurement of colonies parameters. Colonies were identified using the module Identify Primary Object with three classes intensity threshold: foreground, middle and background. Middle class pixels were then categorized as background, to avoid overestimation of the colony area.

### Oxygen consumption rate (OCR)

OCR was determined by direct measurement with a SeaHorse Extracellular Flux Analyzer XpE instrument (Agilent Technologies). OCR for the mitochondrial stress test was determined following the manufacturer’s instructions. OCR of HeLa stable clones was measured at baseline and after the addition of the stressors oligomycin to evaluate ATP production, FCCP to measure the maximal respiration and rotenone and antimycin A for the spare capacity calculation. Time and type of stressor administration are indicated in the graph (Fig. 3a). For statistical analyses, all OCR values were normalized with those of SCR.

### Statistical analysis

Statistical analysis was performed using the Microsoft Excel. One-way ANOVA was used for three group comparisons and Student’s t-test was used for two group comparisons. *p* values of less than 0.05 were considered as significant, while values less than 0.01 or lower were considered as highly significant.

## Results

### Mitochondrial accumulation of APE1 in grades 1 and 2 prevents mtDNA damage

**Fig. 1:**
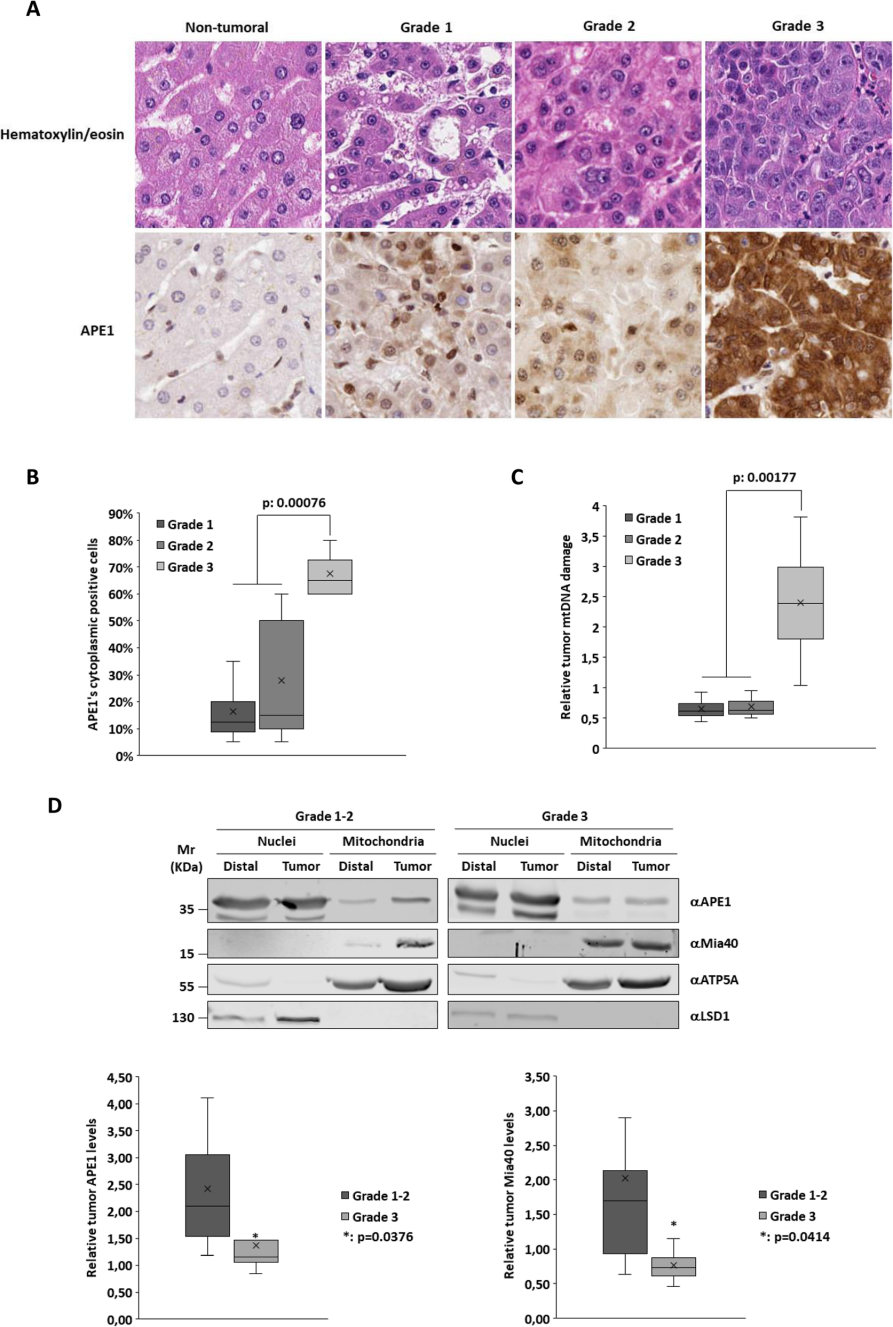
HIC, Western blot analysis and mtDNA damage quantification in human HCC samples. **a** Representative images of non-tumor, Grades 1, 2 and 3 samples stained with hematoxylin/eosin (20x) (*upper panel*) and APE1 (20x) (*lower panel*). **b** Graph showing the percentage of APE1’s cytoplasmic positive cells in tumor tissues. **c** Graph showing the relative levels of mtDNA damage of tumor tissue with respect to the distal non-tumor area of the same patient. **d** Representative Western blot analyses of nuclear and mitochondrial protein extracts of Grades 1–2 and Grade 3 tumor samples (*upper panel*). The graphs (*lower panel*) show the relative amounts of Mia40 and APE1 in the tumor tissues (Tumor) with respect to the distal non-tumor area (Distal) of the same patient. Anti-LSD1 antibodies and anti-ATP5A proteins were used as nuclear and mitochondrial markers, respectively. Full-length blots are presented in Supplementary Figure 1. (*: *p* < 0.05)

IHC analysis was performed to determine the tumor grade for each patient and to evaluate whether APE1 expression and localization changed during the staging. As seen in Fig. 1a, APE1 shows a weak/moderate cytoplasmic positivity in Grades 1 and 2 and a strong cytoplasmic expression in Grade 3. Statistical analysis confirmed this trend, showing a higher number of cytoplasmic positive hepatocytes in Grade 3 compared with lower grades (Fig. 1b). Next, we measured the relative levels of mtDNA damage of the tumor tissue as compared to the non-tumor area of the same patient. In Grades 1 and 2, mtDNA was less damaged in the tumor compared to the distal tissue, while in Grade 3 mtDNA the number of lesions was significantly higher (Fig. 1c). Based on this data, we decided to verify whether APE1’s cytoplasmic positivity observed in IHC analysis accounted for the protein’s accumulation within the mitochondrial compartment and therefore justified the lower levels of mtDNA damage measured. Western blot analyses of nuclear (NCE) and mitochondrial (MCE) protein extracts from non-tumor (Distal) and HCC (Tumor) confirmed that Grades 1 and 2 were characterized by a significantly higher amount of APE1 in mitochondria in the tumor compared to the distal area of all patients, while Grade 3 showed the opposite pattern (Fig. 1d and Supplementary Figure 1). Altogether, these data suggest that APE1’s cytoplasmic positivity in Grades 1 and 2 reflects an increased amount of mitochondrial APE1, explaining the lower levels of mtDNA damage observed. In contrast, the cytoplasmic positivity in Grade 3 is not associated with APE1’s mitochondrial accumulation, thus accounting for the higher number of mtDNA lesions in that grade.

In a previous study we identified the MIA pathway as responsible for the mitochondrial translocation of APE1 [25]. To verify if mitochondrial accumulation of APE1 was somehow associated with an upregulation of its import pathway, we analyzed the amount of Mia40, the main player in this pathway (Fig. 1d). The trend was similar to that of APE1, confirming the direct correlation between Mia40 and APE1 expression and the enhanced mitochondrial DNA damage repair capacity in lower tumor grades.

### Expression of APE1 only in mitochondria is sufficient to sustain cell growth and cellular respiration

**Fig. 2:**
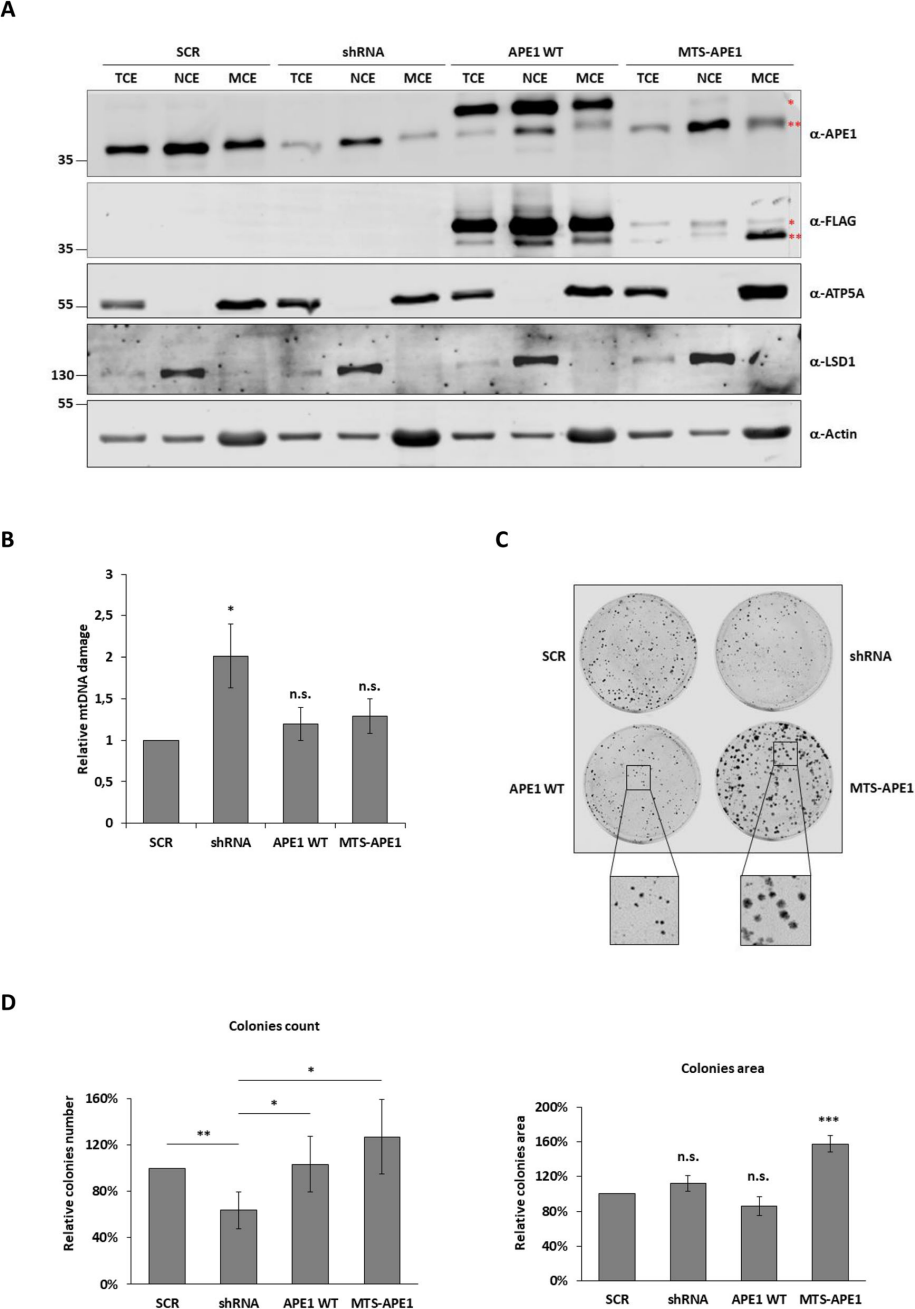
Characterization of APE1 WT and mitochondrial targeted (MTS-APE1) clones. **a** Western blot analysis of total (TCE), nuclear (NCE), and mitochondrial (MCE) protein extracts of control (SCR), APE1 shRNA (shRNA), APE1 WT, and MTS-APE1 HeLa clones. Endogenous APE1 is efficiently silenced by a shRNA clone, while ectopic 3xFLAG-tagged APE1 is present only in APE1 WT and MTS-APE1 clones. While in the APE1 WT clone the ectopic protein localizes into both the nuclear and mitochondrial compartments, in MTS-APE1 clones the signal is mostly present in the MCE. Moreover, ectopic MTS-APE1 pre-protein (*) is processed into the mitochondrial matrix where the MTS is removed (**), leaving the active APE1 form. Anti-LSD1 antibodies and anti-ATP5A proteins were used as nuclear and mitochondrial markers, respectively, while anti-Actin as loading control. Full-length blots are presented in Supplementary Figure 2. **b** mtDNA damage was quantified in each clone. The silencing of APE1 causes a significant increase in the damage detected, while the re-expression of the ectopic APE1 WT or MTS-APE1 restores the basal mtDNA damage. The error bar represents the standard deviation of three independent experiments. (*: p < 0.05). **c** Representative images of clonogenic assay on APE1 shRNA and stable KI clones. In the zoomed squares it is possible to appreciate the size difference between APE1 WT and MTS-APE1 colonies. **d** The graphs report the relative number of colonies (*left panel*) and the colonies’ dimension (*right panel*) of shRNA, APE1 WT, and MTS-APE1 clones compared to the control clone (SCR). Data reported are the mean of four independent biological replicates. (*: p < 0.05; **: *p* < 0.01; ***: *p* < 0.001)

Having established a correlation between the tumor stages, the amount of APE1 in mitochondria, and the extension of mtDNA damage, we decided to fully investigate the role of APE1’s mitochondrial form in HCC progression. Initially we tried to develop on hepatic cell lines JHH6 and Huh7 a conditional knock-down of APE1 by RNAi technology, as we already did in HeLa cell line [40], to study molecular changes associated to loss of APE1 functions. Unfortunately, we have not been able to select any positive clones, and therefore we decided to developed a stable cell line where APE1’s NLS has been substituted by the MTS of MnSOD2 to drive all ectopic protein into the mitochondrial matrix (MTS-APE1). As a control we used a knock-in (KI) clone expressing wild-type APE1 (APE1 WT). The expression of both ectopic proteins was performed on the background of a stable inducible APE1 silencing clone already developed in our laboratory [40]. Scramble control (SCR) and inducible shRNA (shRNA) clones were also included in our analyses. The expression levels and localization of ectopic 3X-FLAG tagged proteins were confirmed by Western blot analyses (Fig. 2a and Supplementary Figure 2). Total (TCE), nuclear (NCE), and mitochondrial (MCE) protein extracts were isolated as described in the materials and methods section. Fifteen μg of total, 15 μg of nuclei, and 40 μg of mitochondria protein extracts were loaded on SDS-PAGE, transferred to nitrocellulose membrane, and analyzed to evaluate the expression of endogenous (α-APE1) and ectopic (α-FLAG) APE1. Doxycycline treatment efficiently silenced endogenous APE1. In APE1 WT clones, ectopic protein is present in both the nuclear and mitochondrial compartments, while in MTS-APE1 clones the mitochondrial targeting sequence efficiently drives all ectopic protein into the mitochondrial matrix (Fig. 2a). This cellular model allowed us to discriminate between the mitochondrial form of APE1 and its nuclear counterpart.

Nowadays, the only biological function described for APE1 in mitochondria is its role in the base excision repair (BER) pathway. To support the validity of our cellular model for studying the relevance of the mitochondrial APE1 form in tumor cells, we measured the levels of mtDNA damage. As previously reported, loss of APE1 expression led to increased levels of mtDNA damage, while the re-expression of APE1 WT rescued the phenotype [25]. Remarkably, re-expression of the MTS-APE1 also resulted in reducing the levels of mtDNA damage, confirming the validity of the model for studying the role of mitochondrial APE1 in cell physiology (Fig. 2b).

To evaluate if the expression levels of APE1 in mitochondria may influence cell phenotype, we performed a clonogenic assay (Fig. 2c). This assay is aimed to estimate the ability of a single cell to form a colony and consequently to evaluate its proliferation ability. The same number of cells from each clone (500 cells/petri) was plated and grown for 10 days in the presence of doxycycline. Then, colonies were stained with methyl violet and the number of colonies and their area were counted using CellProfiler (Fig. 2c). As we already reported in another manuscript [40], and similar to the previously reported evidence using different cell lines [45], clonogenic assay showed a strong inhibition of cell growth in the silenced clone. The growth inhibition was efficiently rescued by the expression of APE1 WT [41]. Interestingly, we also observed a complete rescue of the phenotype in the MTS-APE1 clone, suggesting a fundamental role of the mitochondrial form of APE1 in cell growth processes (Fig. 2d, *left panel*). Next, we analyzed the dimension of the colonies, observing that when APE1 is driven into mitochondria, colonies are significantly bigger compared to both silenced (shRNA) or APE1 overexpressing cells (APE1 WT) (Fig. 2d, *right panel*), suggesting that APE1 expression levels in mitochondria may impact cell growth during the early phases of tumor progression.

**Fig. 3:**
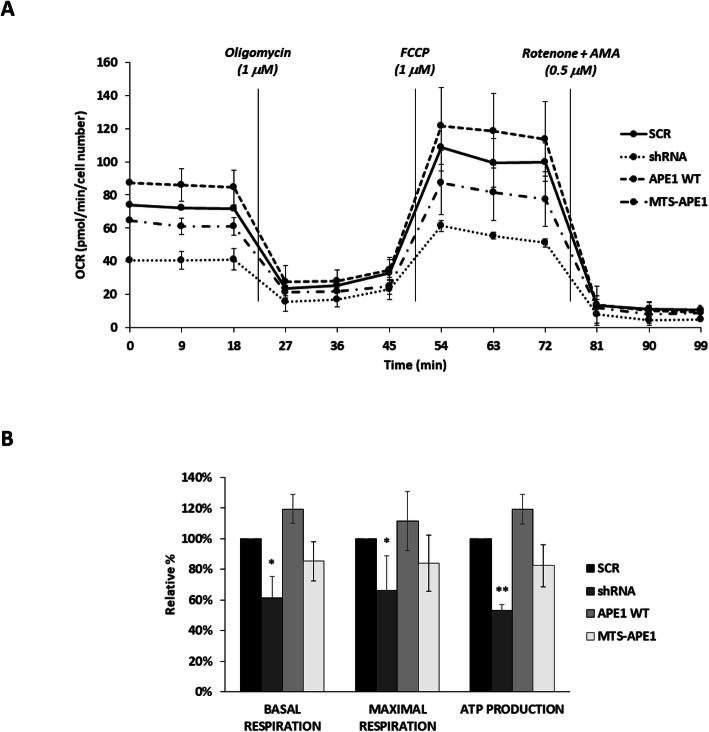
Effect of APE1 silencing on mitochondrial respiratory parameters. **a** Profiles of mitochondria bioenergetics measurements measured with Seahorse. Time and type of stressor administration are indicated. **b** The graph displays the relative average (±SD) basal and maximal respiration, as well as ATP production of HeLa clones silenced for APE1 expression (shRNA) and after re-expression of APE1 in all cells (APE1 WT) or only into the mitochondrial matrix (MTS-APE1). Data reported are the mean of four independent biological replicates. (*: p < 0.05; **: p < 0.01)

Because the localization of APE1 in mitochondria allowed the protein to modify the colonies’ phenotype, thus improving their proliferation, we decided to further investigate if mitochondria physiology was affected by mitochondrial expression levels of APE1. For this purpose, the Oxygen Consumption Rate (OCR) of APE1 shRNA and KI clones was measured using Seahorse extracellular flux analyser (Fig. 3a). Loss of APE1 expression determined significant reduction of both the basal and maximum respiration levels, as well as ATP production (Fig. 3b). All parameters were efficiently rescued in APE1 WT clones. The MTS-APE1 cells’ ability to re-establish SCR levels of OCR and ATP production was impressive (Fig. 3b). Our data show that the presence of APE1 within the mitochondrial matrix is enough to improve all the considered parameters.

## Discussion

The APE1 protein’s importance in maintaining cell homeostasis by playing a major role in the BER pathway and thereby preserving the integrity of nuclear DNA has been broadly discussed [41, 42, 45–50]. Because of its crucial role, APE1 has been described as one of the factors involved in the development of numerous cancer types, and its overexpression has been associated with poor prognosis for patients [51–54]. However, until recently, researchers mainly focused on the nuclear fraction of APE1, despite the knowledge that it also may localize in the mitochondrial compartment [20, 21]. Indeed, cytoplasmic re-localization of APE1 has been found to be associated with a higher tumor aggressiveness and a poorer prognosis for patients with HCC [38]. In a previous work, we demonstrated that the MIA pathway is involved in the translocation of APE1 into the mitochondrial compartment, contributing to the maintenance of mitochondrial genome stability [25]. By using siRNA technology on HeLa cells and JHH6 hepatic cell line we proved that loss of Mia40 expression determined an increase of mtDNA damage under oxidative stress conditions. Moreover, analysing the expression levels of Mia40 and APE1 in hepatic cell lines JHH6 and Huh7 we demonstrated the role of the MIA pathway in the translocation of APE1 and that loss of Mia40 expression negatively affects the mitochondrial levels of APE1 protein [25]. Moreover, a recent manuscript by Li et al. shows how overexpression of APE1 and Mia40 enhances cisplatin resistance and autophagy of A549 cells [55].

In the present study, we further investigated the significance of mitochondrial APE1, particularly in the context of HCC development. As an in vivo model we analysed a cohort of patients affected by HCC who underwent surgical resection. Diagnosis and tumor grade were confirmed by IHC analyses. We also evaluated APE1 expression and localization, revealing that the increased cytoplasmic positivity observed in Grades 1 and 2 accounted for the higher amount of mitochondrial APE1 and the lower levels of mtDNA damage in the tumor tissue with respect to the distal area measured. In accordance with our model and the data reported in literature, the expression levels of Mia40 were also found to be upregulated, justifying the higher amount of APE1 observed in the mitochondrial compartment. In contrast, compared with lower-grade patients, Grade 3 patients showed higher numbers of cytoplasmic positive hepatocytes, reduced expression levels of APE1 and Mia40, and a significantly higher number of DNA lesions. In the cohort of patients analyzed there have been eight recurrences, one of which was previously classified as Grade 3 and seven as Grade 2. In six of those Grade 2 patients the levels of mtDNA damage were lower in the tumor tissue respect to the distal area and the percentage of APE1 cytoplasmic positive cells was between 40 and 60%. This observation further support the role of mitochondrial APE1 in tumor development and cellular migration processes.

To better understand the role of APE1 mitochondrial fraction, we generated a stable cell line where APE1’s NLS has been substituted by the MTS of MnSOD2 to drive all ectopic protein into the mitochondrial matrix (MTS-APE1). Remarkably, the presence of APE1 within the mitochondrial matrix was sufficient to rescue respiration, cell viability, and proliferation as well as mtDNA levels, all of which were negatively affected by APE1’s loss of expression.

**Fig. 4:**
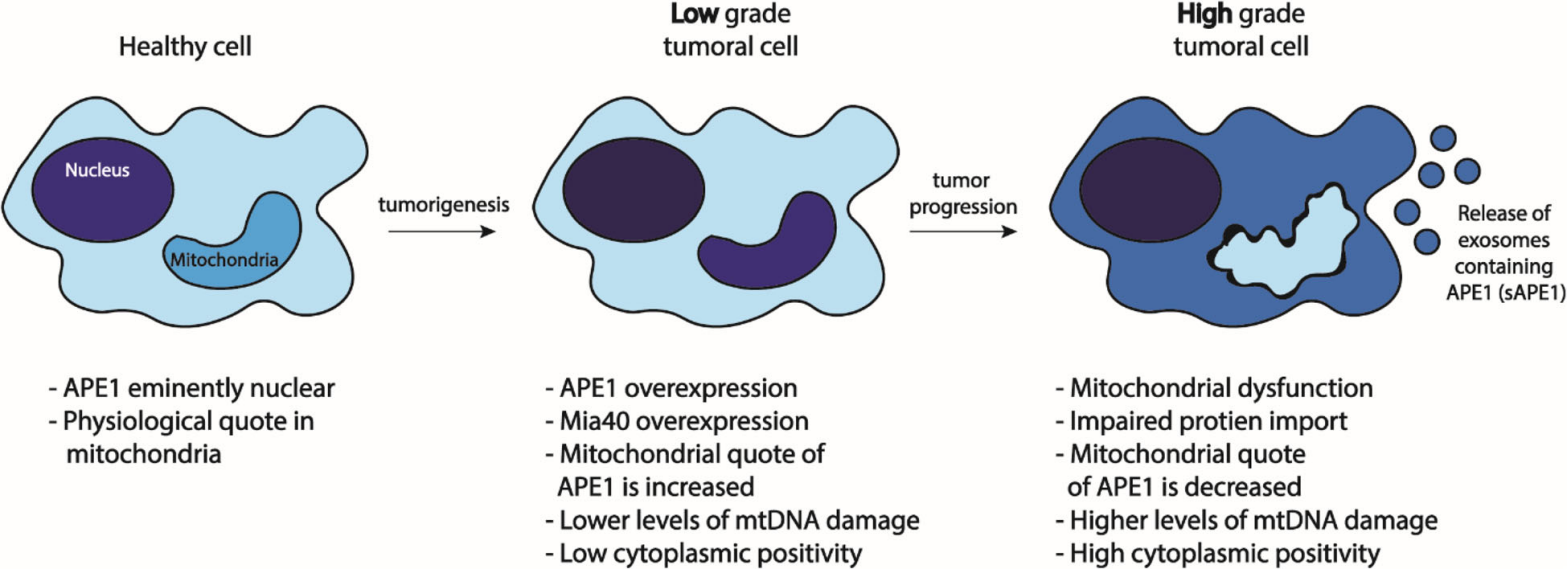
Role of mitochondrial APE1 in HCC tumor progression. In healthy cells, APE1 localization is mainly nuclear. Nevertheless, a physiological amount of the protein is also present in mitochondria (healthy cell). During tumorigenic processes and in low grade tumors, APE1 is overexpressed. Increased expression of its mitochondrial transporter Mia40 determines increased levels of mitochondrial APE1, which determines a higher rate of mtDNA repaired. In high grade tumors, APE1 delocalizes from nuclei to cytoplasm; mitochondria show dysfunction, the protein import is impaired, and less APE1 is detected in the organelle. At this final stage, exosomes containing APE1 have been detected in serum (sAPE1)

Our data suggest that during the early phase of tumor development (Grades 1 and 2), while cell proliferation is at its highest [56, 57] and the accumulation of mutations is relatively low [57], the high-energy demand to sustain an enhanced cell metabolism requires increased ATP production via oxidative phosphorylation generating more ROS. Therefore, maintaining mitochondrial stability is crucial, and this justifies the increased amount of APE1 present in this organelle in Grades 1 and 2 patients. In these stages, overexpression of Mia40 increases the import of APE1, contributing to the maintenance of mtDNA integrity. In contrast, during the latest tumor stage (Grade 3), when the overall cell fitness is impaired by accumulation of the mutations, the efficiency of the protein transport into the mitochondria can be significantly decreased. This explains why the cytoplasmic positivity in Grade 3 is not associated with APE1’s mitochondrial accumulation, and accounts for the higher number of mtDNA lesions measured in the tumor tissue with respect to the distal area (Fig. 4). In accordance with our data, in a recent manuscript Pascut et al. detected the presence of APE1 in the serum of HCC patients, suggesting this parameter as a possible biomarker for tracking cancer progression [58].

## Conclusions

Results obtained by analysing the patients’ cohort are in agreement and extend with an in vivo model the previously published data correlating tumor development with APE1 expression and localization. To our knowledge, this is the first study on patients’ samples where mitochondrial APE1 expression has been evaluated and correlated with the levels of mtDNA damage and the tumor stage. Our data clearly indicate a leading role of the mitochondrial form of APE1 in the early stages of cancer development and the relevance of the non-nuclear fraction of APE1 in the disease progression. In our model, the mitochondrial form of APE1 is essential in protecting the mtDNA and in this way preserving the cells’ ability to actively proliferate (Fig. 4). Our study has also confirmed overexpression of Mia40 and the role of the MIA pathway in the APE1 import process. Overall, our findings clearly illustrate that inhibition of the APE1 transport by blocking the MIA pathway could become an alternative therapeutic approach. This procedure would trigger reduction of the mitochondrial metabolism, which would prevent efficient repair of mtDNA.

## Supplementary information

**Additional file 1.**

**Additional file 2.**

## Data Availability

The datasets used and analyzed during the current study are available from the corresponding author on reasonable request.

## Abbreviations

8-oxoG: 8-oxoguanine
APE1: Apurinic/apyrimidinic endonuclease 1;
ATP5A: ATP synthase subunit alpha
BER: Base Excision Repair
ETC: Electron Transport Chain
FCCP: Carbonyl cyanide-4-(trifluoromethoxy)phenylhydrazone
HCC: Hepatocellular Carcinoma
IHC: Immunohistochemistry
IMS: Intermembrane Space
LSD1: Lysine-specific histone demethylase 1
MIA: Mitochondrial IMS Assembly
NLS: Nuclear Localization Sequence
OCR: Oxygen Consumption Rate
ROS: Reactive Oxygen Species
SCR: Scramble
TOM: Translocase of the Outer Membrane.
