# Supplementary figures and images for: Mitochondrial apurinic/apyrimidinic endonuclease 1 enhances mtDNA repair contributing to cell proliferation and mitochondrial integrity in early stages of hepatocellular carcinoma
Source: BMC Cancer. 2020 Oct 7;20:969. doi: 10.1186/s12885-020-07258-6 (PMC7542375; doi:10.1186/s12885-020-07258-6)

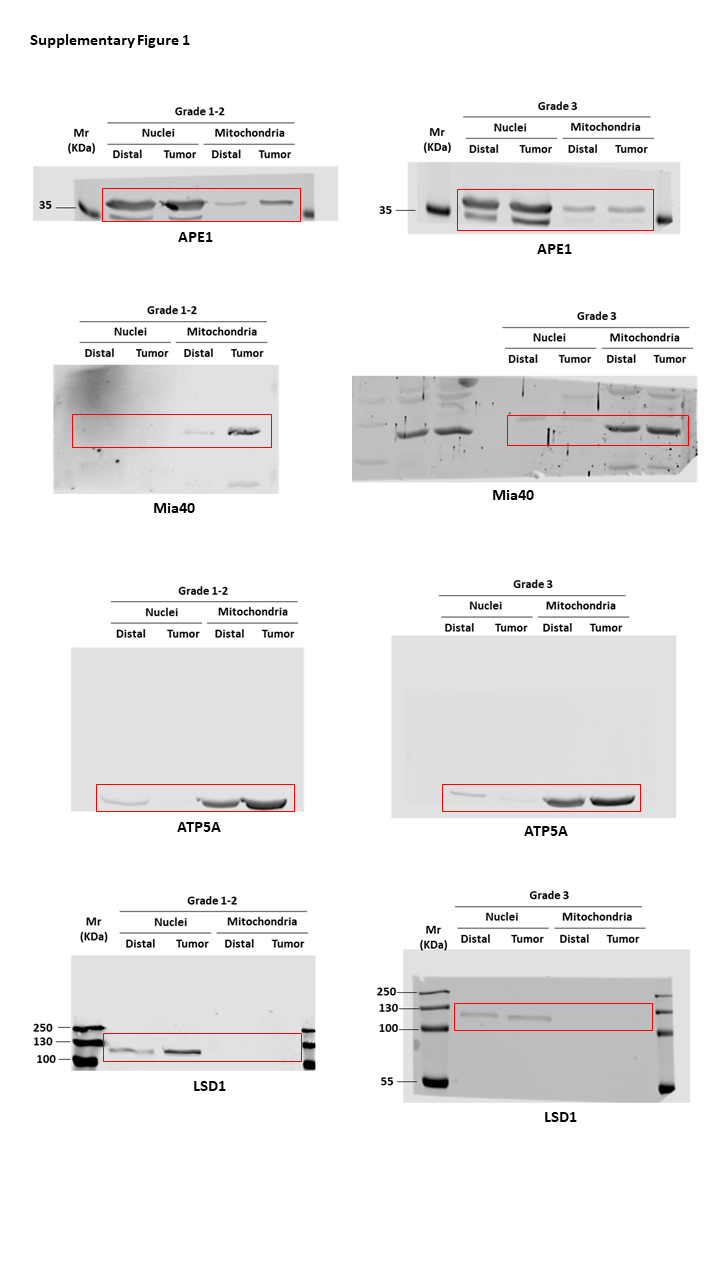

Supplement: Supplementary file 1 — Additional file 1. [file 12885_2020_7258_MOESM1_ESM.tif]

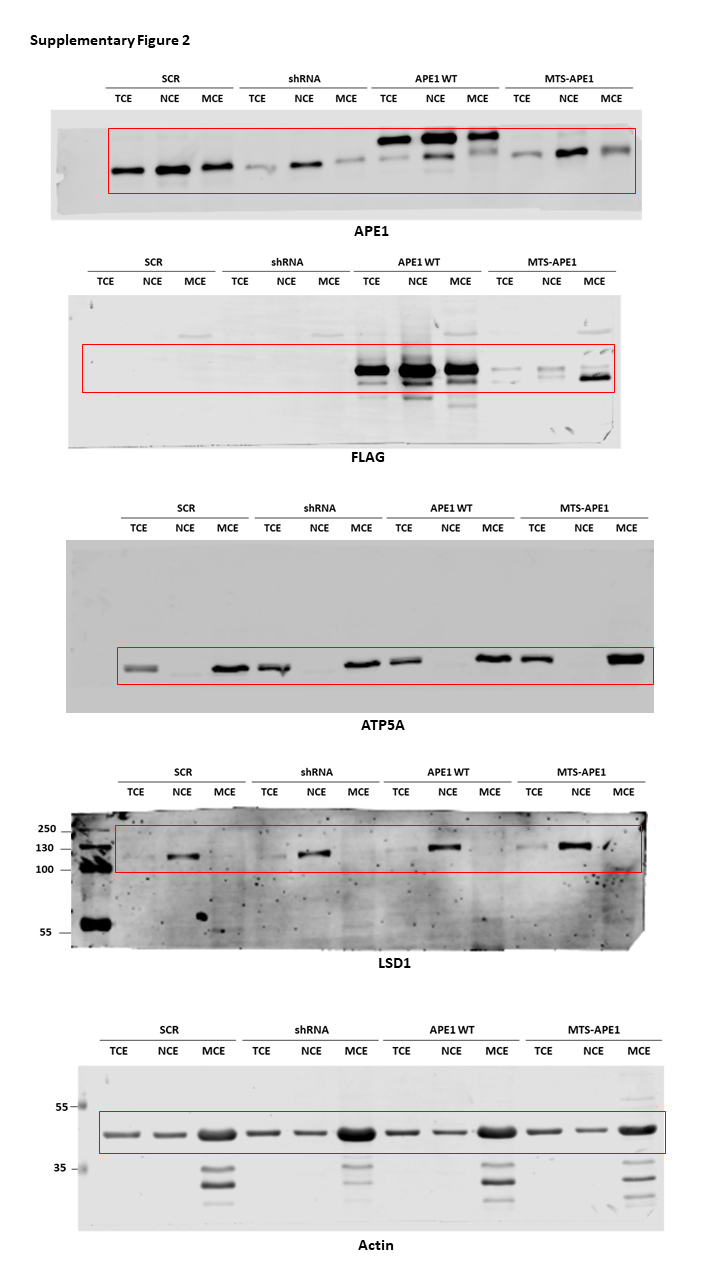

Supplement: Supplementary file 2 — Additional file 2. [file 12885_2020_7258_MOESM2_ESM.tif]
